# Comments on Study of “Performance of 18F-DCFPyL PET/CT in Primary Prostate Cancer Diagnosis, Gleason Grading and D'Amico Classification: A Radiomics-Based Study”

**DOI:** 10.1007/s43657-023-00143-9

**Published:** 2023-12-04

**Authors:** Michael C. Kreissl

**Affiliations:** Division of Nuclear Medicine, Department of Radiology and Nuclear Medicine, Otto von Guericke University, Leipziger Str. 44, 39120 Magdeburg, Germany

Prostate cancer remains one of the most prevalent malignancies in men globally (Preisser et al. 2020). Accurate diagnosis and differentiation of the disease are paramount for effective treatment planning and improved patient outcomes (Hsieh et al. 2022). Traditionally, the diagnosis of prostate cancer heavily relied on invasive biopsy procedures, which, although effective, are associated with potential complications and discomfort for patients. With the advancements in medical imaging, positron emission tomography/computed tomography (PET/CT) using prostate-specific membrane antigen (PSMA) tracers has emerged as a revolutionary diagnostic tool (Obek et al. 2017). This technique, often termed as 'virtual biopsy', provides a non-invasive alternative to traditional biopsy. Baseline PSMA PET/CT offers detailed and precise imaging of prostate lesions, allowing clinicians to pinpoint not only the location but also the potential aggressiveness of the tumor. The capability of PSMA PET/CT to bind specifically to PSMA-expressing cells gives it a unique edge in differentiating between benign and malignant prostate lesions. This specificity also aids in identifying metastatic or recurrent diseases, often before they become evident on conventional imaging modalities (Papp et al. 2021).

In this comprehensive investigation, a patient cohort, consisting of 138 individuals exhibiting clinical indicators suggestive of prostate carcinoma, underwent detailed imaging studies using piflufolastat F-18 (^18^F-DCFPyL) PET/CT. This imaging modality captured a wide spectrum of prostate pathologies, encompassing benign hyperplasia, benign inflammatory processes, and a range of malignant neoplasms, each varying in their gleason score (GS) and D'Amico scores. Beyond just providing the conventional PET metrics, which include but are not limited to standard uptake value max (SUVmax), standard uptake value mean (SUVmean), standard uptake value ratio (SUVR), total lesion PSMA (TL-PSMA), and total volume  PSMA (PSMA-TV), this advanced imaging modality yielded granular tumor texture attributes, contextualized against the background data. Li et al. leveraged an advanced machine learning algorithm, meticulously rooted in the radiomics of ^18^F-DCFPyL positron emission tomography/computed tomography (PET/CT) imaging. This algorithm was meticulously developed with the aim of providing an innovative, non-invasive strategy for the efficacious stratification of prostate lesions (Lambin et al. 2017; Perandini et al. 2016). Its capabilities extend from differentiating benign from malignant prostate lesions, pinpointing high-grade pathological prostate neoplasms (with a Gleason score exceeding seven), to identifying prostate malignancies associated with an elevated D'Amico risk score.

This approach was pivotal in discerning the intricate interplay between in vivo attributes, patient demographics, and their respective significance in prognosticating malignancies, especially those of elevated risk. Through a systematic amalgamation and deployment of these weighted features, they succeeded in formulating robust prognostic models. These models catered to a spectrum of diagnostic challenges, from general malignancy diagnosis (Mm) to high-grade prostate carcinoma identification (Mgs) and risk stratification of high clinical-risk prostate pathologies (Mamico).

Ensuring the validity and applicability of the findings, these formulated models underwent rigorous validation via a Monte Carlo cross-validation paradigm. The results were promising. For the entire subset of primary prostate carcinoma subjects, the Monte Carlo metrics reflected compelling efficacy of the models with AUC values of 0.97 for Mm, 0.73 for Mgs, and 0.82 for Mamico. Beyond mere numbers, these findings underscore the transformative potential of ^18^F-DCFPyL PET/CT radiomics. It signifies a paradigm shift, enabling clinicians to discern between benign and malignant prostate tumors, as well as to identify high-risk neoplasms, all while negating the invasiveness of traditional biopsy approaches.

This innovation's implications are vast in the clinical landscape of prostate patient management. Particularly, when it comes to initial assessments and baseline evaluations, the diagnostic accuracy this method offers is unparalleled. It acts as a beacon, illuminating clinical therapeutic strategy formulations, and more importantly, mitigating potential physiological and financial adversities induced by redundant or unnecessary biopsies.

This study, born from a collaboration of clinical radiologists and academia spanning from China to Europe, may contribute significantly to a better management of patients with (suspected) prostate cancer. The integration of machine learning has paved the way for a unified, global benchmark for prostate patient diagnosis and risk stratification, ensuring that patients are spared from physical harm during their diagnostic journey. The methodology, being avant-garde, methodologically sound, systematized, and embedded with clinical pragmatism, is positioned for global acceptance and adoption. Its merits, beyond doubt, warrant recognition and integration into clinical guidelines. However, a transparent acknowledgment of this study's limitations includes its sample size constraints and the limited number of associated research institutions. These aspects warrant attention and amplification in forthcoming research endeavors.

The clinical significance of this 'virtual biopsy' technique is profound. Early PSMA PET/CT facilitates the formulation of more personalized treatment plans based on the precise location and extent of the disease. Furthermore, by reducing the need for invasive biopsy procedures, patients are spared potential complications such as infection, bleeding, and discomfort. The non-invasive nature of the PSMA PET/CT not only enhances patient comfort but also expedites the diagnostic process, ensuring that therapeutic interventions can commence promptly. All in all, the advent of an initial PSMA PET/CT as a 'virtual biopsy' tool in the realm of prostate cancer diagnosis and differentiation has transformed the clinical landscape. By providing precise, detailed, and non-invasive insight into prostate pathology, it plays a pivotal role in improving patient outcomes and revolutionizing prostate cancer management.

## Data Availability

Not applicable.
